# Supplementary material for: Protocol for Designing New Functional Food with the Addition of Food Industry By-Products, Using Design Thinking Techniques—A Case Study of a Snack with Antioxidant Properties for Physically Active People
Source: Foods. 2021 Mar 24;10(4):694. doi: 10.3390/foods10040694 (PMC8064058; doi:10.3390/foods10040694)
Supplement: Supplementary file 1 [file foods-10-00694-s001.pdf]

foods

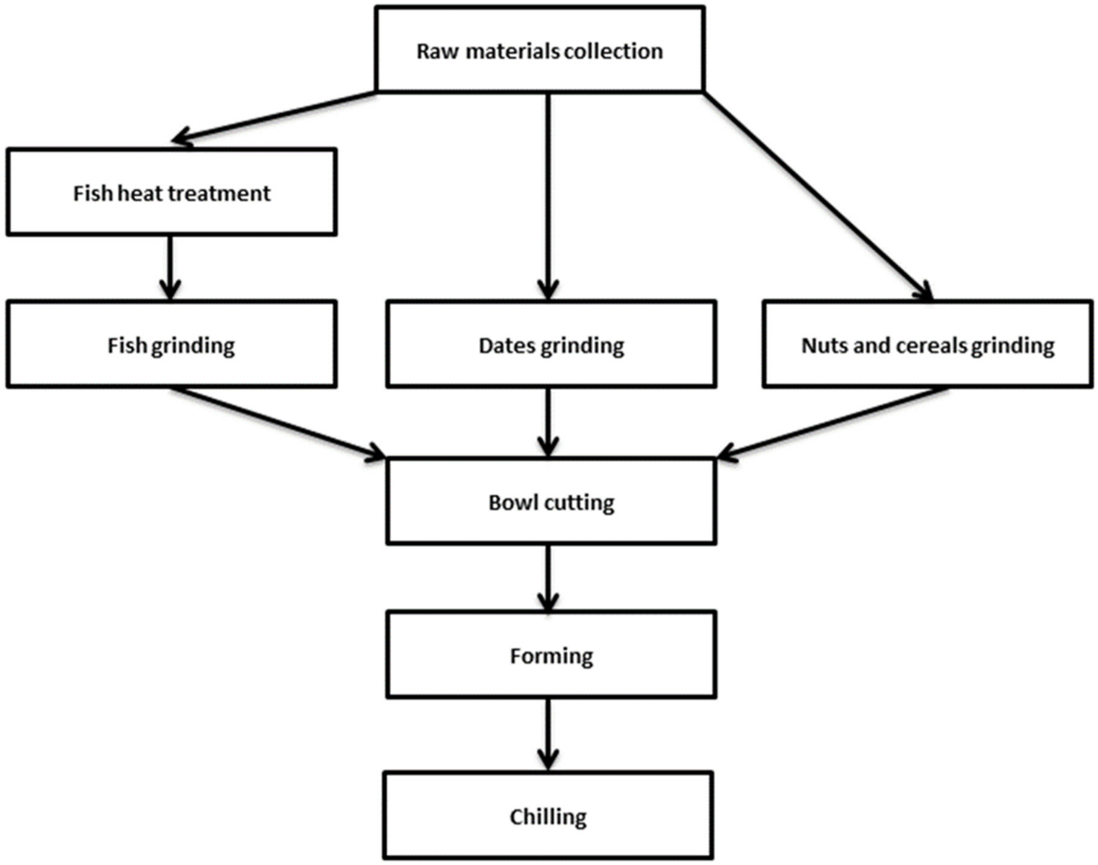

Figure S1. The production process of the first version of the date bars with carp meat and CSGH.

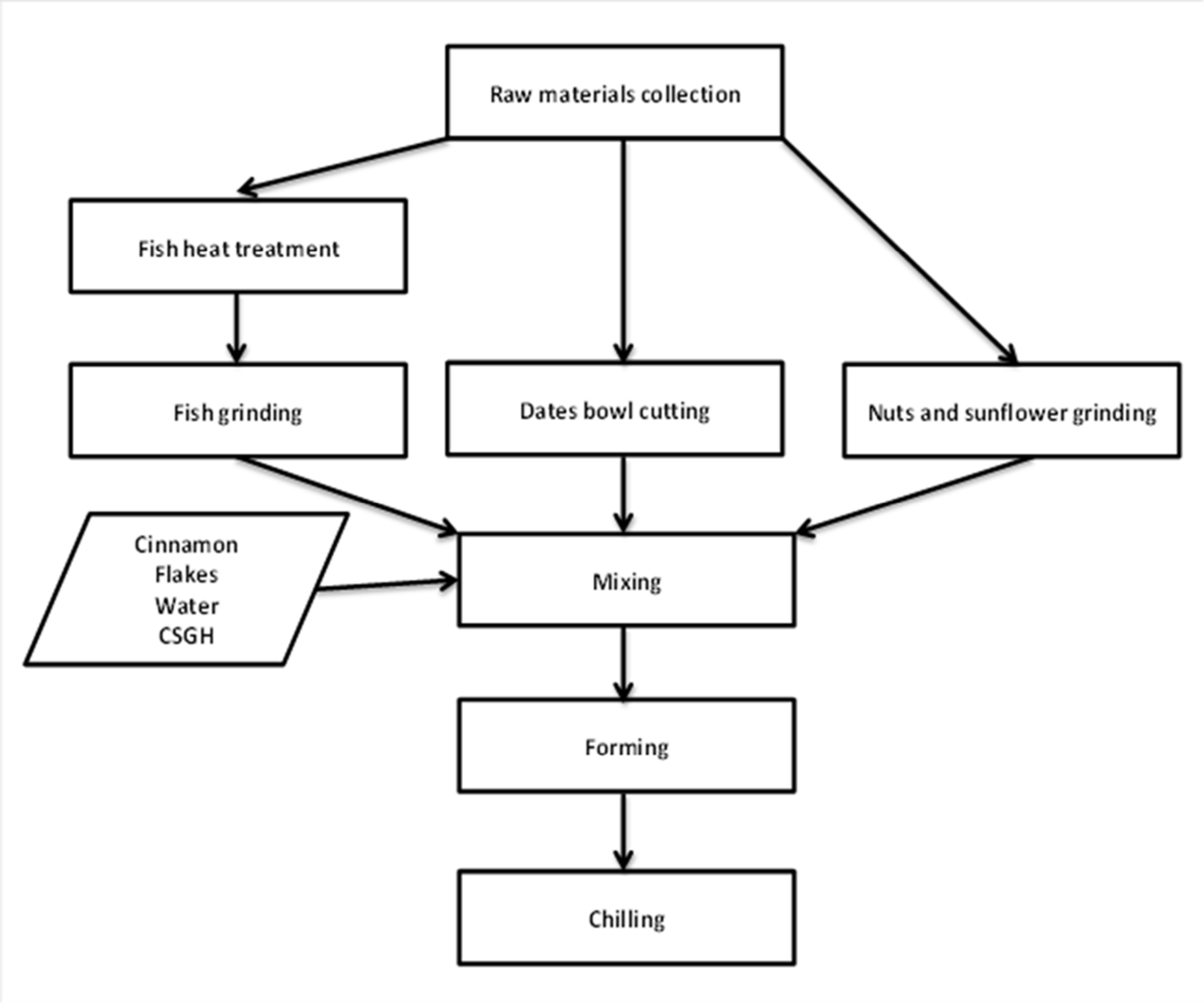

Figure S2. The production process of the final version of the date bars with carp meat and CSGH.

Table S1. The recipe of the first and final version of the date bar with carp meat and CSGH.

Ingredient	First Recipe [%]	Final Recipe [%]
Carp meat	40.00	29.00
Dried dates	29.50	36.00
Buckwheat flakes	21.00	25.00
Pecan nuts	3.00	3.25
Sunflower seeds	2.50	2.25
Water	2.50	3.00
CSGH	1.00	1.00
Cinnamon	0.50	0.50

Detailed information about methodology of chemical analysis

Qualitative Index		Product Evaluation 5 (High)	4 (Good)	3 (Satisfactory)	2 (Unsatisfactory)	1 (Unacceptable)
General appearance	0.15	Even shape, cohesive mass, non-stratifying, no	Acceptable unevenness in shape, cohesive mass,	Slightly deformed shape, slightly compact	Deformed shape, hard or crumbling mass, too large or	Shapeless, crushed or melting mass,

		cracks, visible pieces of dried fruit and nuts	partly stratifying, visible pieces of dried fruit and nuts	or slightly crumbling mass, visible pieces of dried fruit and nuts	completely invisible dried fruit and nut fragments	fragments of fruit and nuts not visible
Colour	0.1	Golden-brown, slightly shiny surface, with visible pieces of dried fruit and nuts, characteristic in colour	Golden-brown, matte surface with visible pieces of dried fruit and nuts, characteristic in colour	Brown, matt, uniform on the entire surface, only slightly visible pieces of dried fruit and nuts	Brown or brown-gray, matte, uniform on the entire surface, pieces of dried fruit and nuts not detectable	Inappropriate, uncharacteristic for fruit bars with nuts and dried fruits
Smell	0.1	Specific for bars with nuts and dried fruits, intense, pleasant, different noticeable hints, incl. cinnamon or gingerbread, but without undesirable hints of spoilage	Specific, not pure, but still noticeable hints of dried fruit and nuts and, but without undesirable hints proving spoilage of the product	Peculiar, not pure	Slightly altered, foreign hints perceptible	Unspecific or no smell, foreign, characteristic of spoilage
Texture in mouth (take a bite of the bar and place it between the tongue and the palate, moving the tongue, chew up the sample then masticate it several times)	0.15	Specific mass but not sticky to the palate, noticeable fragments of dried fruit and nuts, suitably soft and juicy, not requiring much chewing	mass, slightly sticky to the palate, noticeable fragments of dried fruit and nuts, a bit too soft or a bit too hard, does not require much chewing	Mass crumbling in the mouth, noticeable fragments of dried fruit and nuts, too soft or too hard, a bit too dry or too fatty, slightly chewy	Crumbling mass in the mouth, palpable pieces of dried fruit and nuts hard and difficult to chew, sandy, melting or hard mass requiring prolonged chewing	Hard, dry mass, hard fruit and nuts that requiring constant chewing and gnawing
Taste	0.2	Moderately sweet, hints of dried fruit and nuts, noticeable hints of, e.g. cinnamon, gingerbread, fish, but no hints indicative of spoilage, such as rancid, sour, fermented taste	A bit too sweet or not enough sweet, hints of dried fruit and nuts, noticeable hints of, e.g. cinnamon, gingerbread, fish, but no hints indicative of spoilage, such as rancid, sour, fermented taste	Too sweet or not sweet enough, taste of fruit and nuts and dried fruits is not noticeable, detectable hints of, e.g. cinnamon, gingerbread, fish, but no hints indicative of spoilage, such as rancid, sour, fermented taste	Too sweet or completely non-sweet, unspecific for fruit and dried fruit, bland, perceptible foreign hints	Completely without specific taste, foreign taste indicative of spoilage
General taste sensation	0.3	Very tasty, very desired	Tasty, desired	Neither tasty nor untasty, neutral	Untasty, undesired	Very untasty, very undesired

1. Amino Acid Composition

The samples were hydrolysed at 110oC in 6M HCl. The hydrolysate was evaporated using the RVO 200A evaporator (Ingos, Prague, Czech Republic) and dissolved in a 2.2 pH buffer. Next, 10 mL of the prepared solution was derivatised by mixing 70 μL of a borate buffer (pH 8.2–9.0) and 20 μL of 6-aminoquinolyl-N hydroxysuccinimidylcarbamate (Waters, Milford, MA, USA) in an acetonitrile solution (3:1, *w/v*). The standards were derivatised identically as the samples. Ami-no acid analysis was performed using the Dionex Ultimate 3000 HPLC system (Thermo Fisher Scientific, Waltham, MA, USA) equipped with an LPG-3400 SD 4-channel gradient pump, WPS 3000 TSL auto-sampler and FLD 3400RS 4-channel fluorescent detector. Analysis was performed on a Nova-Pak C18, 4 μm (150 × 3.9 mm) column (Waters, Milford, MA, USA). The elution buff-ers were (A) acetate-phosphate and (B) 60:40 acetonitrile/water. The separation temperature was adjusted to 37°C, and detection settings were as follows: excitation at 250 nm and emission at 395 nm wavelengths. Quantitative analysis was performed by 1-point calibration with analytical standards (50 pmol for each concentration).

2. B1, B2, B12 Vitamin Content

Detection of B1 and B2 vitamins (mg kg-1 DM) was performed as described in (Starzyńska-Janiszewska, Duliński, Stodolak, Mickowska & Wikiera, 2016) with modifications for thiamine detection by (Mickowska, Dulinski & Kozik, 2000). Separation of riboflavin and vitamin B1 (as thiochrome) was performed on reversed-phase high-performance liquid chromatography (Luna C18, 250 mm × 4 mm i.d., Phenomenex, Torrance, CA, USA), isocratically, with a mobile phase consisting of methanol and 0.05M sodium acetate (30:70 *v/v*) at a flow rate of 1 mL min-1. The fluorimetric detector was set at excitation wavelengths of 366 and 422 nm and emission wave-lengths of 435 and 522 nm for B1 and B2, respectively. Conversion of thiamine to tiochrome was performed post-column, after modification with an oxidising reagent (0.1% potassium hexacy-anoferrate (III) in 12% sodium hydroxide with a peristaltic pump at a 0.2 ml/min flow rate. Vit-amin B12 was determined using the Biopharm test (RIDASCREEN®FAST Vitamin B12 Cat. No. R2103) on an Elisa device.

3. Micro- and Macro-Element Analysis

Selected mineral compounds (calcium, magnesium, potassium, sodium) were analysed using the FAAS flame atomic absorption spectrometric method (Varian AA240FS) - according to PN-EN 15505:2009, and zinc and manganese - PN-EN 14084:2004. Sample mineralisation was conducted in Teflon containers via the high-pressure microwave method (MarsXPres, CEM), with 65% ni-tric acid (Suprapur, MERCK, Cat. No. 1.00441), in the amount of 10 mL/0.5 g per sample. The max. temperature was set to 200 °C, mineralisation time - 40 min. The Schuhknecht and Schinkel buff-er solutions (caesium chlorate and aluminium nitrate in the concentrations 50 g/L and 250 g/L, re-spectively - MERCK, Cat. No. 102037), for potassium and sodium determination as well as the Schinkel buffer solution (caesium chloride and lanthanum chloride in the concentrations of 10g/l – MERCK, Cat. No. 1.16755), for calcium and magnesium analysis were added. The wavelengths for mineral determination were as follows: K-766.5 nm, Ca-222.7 nm, Mg-285.2 nm, Na-589.0, Zn-213.9 nm, Mn-279.5 nm. Accuracy of the applied methods was verified based on certified refer-ence materials (NCS ZC73012 - GSB-5, China National Analysis Center for Iron and Steel, Bei-jing, China). All applied methods were fully validated, checked by internal quality control pro-cedures (according to PN-EN 13804) and interlaboratory/proficiency tests.

4. Chemical Extraction of Antioxidants

Chemical extraction of antioxidants from the innovative snack with CSGH was executed accord-ing to the method described by (Pérez-Jiménez & Saura-Calixto, 2005). To 2.50 g of the homoge-neous sample, 25 mL of methanol/water solution (50:50 *v*/*v*, pH 2) was added and shaken at room temperature for 1 hour. The sample was centrifuged at 2,500 g for 10 minutes. The supernatant was separated and 2.5 ml of acetone/water solution (70:30, *v*/*v*) was added to the remainder and shaken at room temperature for 1 hour. Later, the sample was again centrifuged at 2,500 g for 10 minutes. The acetone supernatant was combined with the methanol solution and filled up to 50 mL.
